# Characterization of the complete mitochondrial genome of the praying mantis *Mekongomantis quinquespinosa* (Mantodea, Mantidae)

**DOI:** 10.1080/23802359.2019.1668737

**Published:** 2019-09-27

**Authors:** Shi Yan, Yuan Zhong Lin

**Affiliations:** Key Lab of Integrated Crop Pest Management of Shandong Province, College of Plant Health and Medicine College, Qingdao Agricultural University, Qingdao, China

**Keywords:** *Mekongomantis quinquespinosa*, praying mantises, mitogenome

## Abstract

The complete mitochondrial genome of the praying mantises *Mekongomantis quinquespinosa* was characterized in this study. The circular molecule is 15388 bp in length (GenBank accession no. MN267041), containing 13 protein-coding genes (PCGs), 2 ribosomal RNA (rRNA) genes, 22 transfer RNA (tRNA) genes. The nucleotide composition is asymmetric (39.0% A, 15.8% C, 9.8% G, 35.4% T), with an overall A + T content of 74.4%. The gene arrangement of *M. quinquespinosa* is identical to that observed in other Mantidae family praying mantises. Ten reading frame overlaps and seven intergenic regions are found in the mitogenome of *M. quinquespinosa*. The phylogenetic relationships based on 13 PCGs show that *M. quinquespinosa* clusters closest to the species of Mantidae.

*Mekongomantis*, established and described a new genus of Mantidae by Schwarz et al. (2018), belong to the Mantidae subfamily Hierodulinae. The samples of a female adult were collected at Jinghong, Xishuangbanna, Yunnan, China (101°25′E, 22°36′N), in 2017, and identified to species by morphology. *Mekongomantis quinquespinosa* captures insect pests as food and is recognized as an important natural enemy for biological control. In this study, samples of *M. quinquespinosa* were stored in the praying mantises specimen room of College of Plant Medicine, Qingdao Agricultural University with an accession number 17-XSBN.

The complete mitogenome of *M. quinquespinosa* is a circular molecule of 15388 bp in length (GenBank under accession no. MN267041), and contains 13 protein-coding genes (PCGs), 22 transfer RNA genes (tRNAs), 2 ribosomal RNA genes (*rrnL* and *rrnS*). The nucleotide composition of *M. quinquespinosa* mitogenome is asymmetric (39.0% A, 15.8% C, 9.8% G, 35.4% T), with an overall A + T content of 74.4%. The AT-skew and GC-skew of this genome were 0.048 and −0.234, respectively. The gene organization of *M. quinquespinosa* is similar to that observed in family Mantidae praying mantises (Ye et al. 2016; Rivera and Svenson 2016; Zhang et al. 2018). Twenty-five genes were encoded on the major strand (J-strand), whereas the others were encoded on the minor strand (N-strand).

The *M. quinquespinosa* mitogenome harbours a total of 53 bp intergenic spacer sequences, which is made up of 7 regions in the range from 7 to 15 bp. Gene overlaps were found at 6 gene junctions and involved a total of 212 bp. The A + T content of the *M. quinquespinosa* mitogenome was 74.4%. The higher A + T content of *M. quinquespinosa* was present in all regions, both genes and noncoding regions. Gln, Glu, and Trp are also the most frequently used. Moreover, the A + T content was also reflected further in the codon usage: the relative synonymous codon usages showed that *M. quinquespinosa* used more NNA and NNT codon. The *rrnL* was 1322 bp in length with A + T content of 78.7%, and *rrnS* was 788 bp in length with A + T content of 75.1%.

Eight PCGs in *M. quinquespinosa* mitogenome start with a typical ATN (ATC, ATT, and ATG) codon, *nad3* and *nad6* begin with TTA, *CoI* uses CTG, *nad1* use GTA and *nad4L* use CAT as initiation codon. Thirteen PCGs stop with a complete termination codon (TAT, TAA, and TAG). Based on the maximum likelihood analyses, we constructed the phylogenetic relationships of *M. quinquespinosa* and 24 other praying mantises based on the 13 PCGs amino acids using RAxML. *Cryptocercus kyebangensis* was used as an outgroup. The results have shown that *M. quinquespinosa* is closely clustered with other species in family Mantidae (Figure 1), which agree with the morphology.

**Figure 1.**
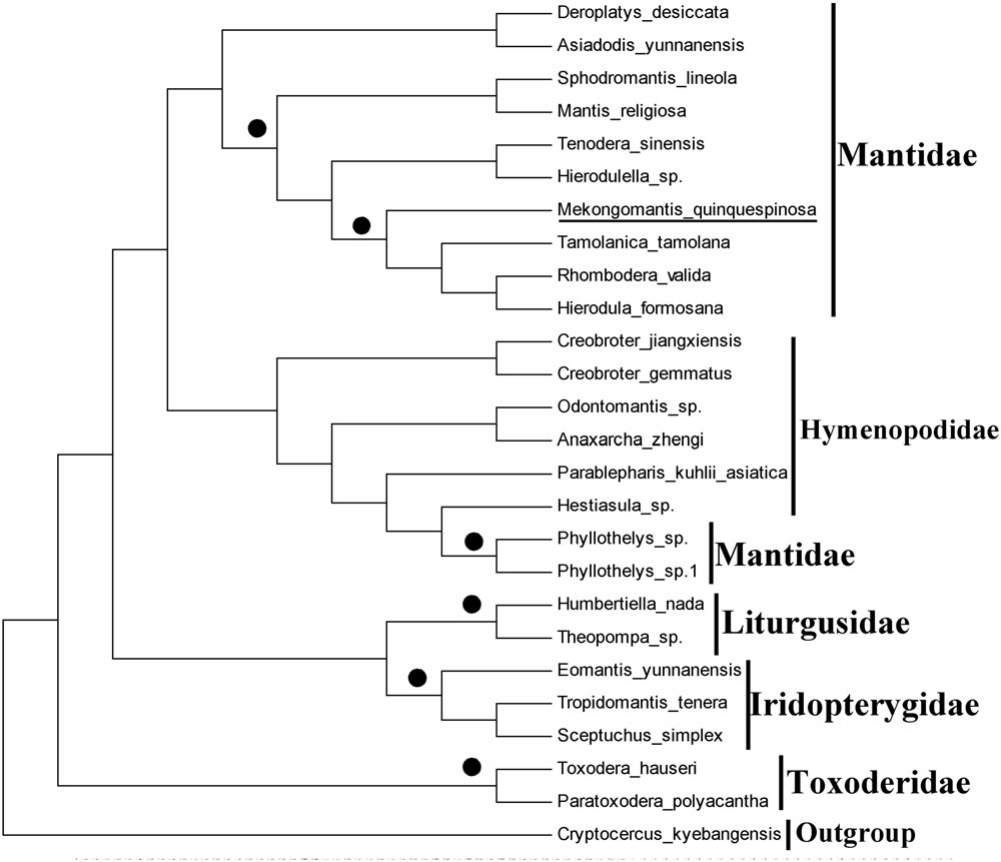
Phylogenetic tree showing the relationship between *Mekongomantis quinquespinosa* (MN267041) and 24 other praying mantises. *Cryptocercus kyebangensis* (KP872847.1) was used as an outgroup. The black circle indicates popularity >90. GenBank accession numbers used in the study are the following: *Rhombodera valida* (KX611804.1), *Hierodula formosana* (KR703238.1), *Tamolanica tamolana* (DQ241797.1), *Sphodromantis lineola* (KY689123.1), *Tenodera sinensis* (KY689132.1), *Hierodulella sp.* (KY689136.1), *Deroplatys desiccate* (KY689113.1), *Mantis religiosa* (KU201317.1), *Sceptuchus simplex* (KY689133.1), *Tropidomantis tenera* (KY689127.1), *Eomantis yunnanensis* (KY689138.1), *Mekongomantis quinquespinosa*, (MN267041), *Theopompa sp.* (KU201314.1), *Humbertiella nada* (KU201315.1), *Anaxarcha zhengi* (KU201320.1), *Odontomantis sp.* ( KY689121.1), *Creobroter gemmatus* (KU201319.1), *Creobroter jiangxiensis* (KY689134.1), *Parablepharis kuhlii asiatica* (KY689117.1), *Hestiasula sp.* (KY689115.1), *Phyllothelys sp.1* (KY689119.1), *Phyllothelys sp.* (KY689129.1), *Toxodera hauseri* (KX434837.1) and *Paratoxodera polyacantha* (MG049920.1). Mantis determined in this study was underlined.
